# Effectiveness of Pharmacological-Based Interventions, Including Education and Prescribing Strategies, to Reduce Subacute Pain After Total Hip or Knee Arthroplasty: A Systematic Review of Randomized Controlled Trials

**DOI:** 10.1093/pm/pnac052

**Published:** 2022-03-24

**Authors:** Shania Liu, Furkan Genel, Ian A Harris, Asad E Patanwala, Sam Adie, Jennifer Stevens, Geraldine Hassett, Kate Luckie, Jonathan Penm, Justine Naylor

**Affiliations:** Faculty of Medicine and Health, School of Pharmacy, The University of Sydney, Camperdown, NSW, Australia; Department of Pharmacy, Prince of Wales Hospital, Randwick, NSW, Australia; St George and Sutherland Clinical School, University of New South Wales, Kogarah, NSW, Australia; Whitlam Orthopaedic Research Centre, Ingham Institute, Liverpool, NSW, Australia; Whitlam Orthopaedic Research Centre, Ingham Institute, Liverpool, NSW, Australia; South Western Sydney Clinical School, Faculty of Medicine, UNSW Sydney, Liverpool, NSW, Australia; Faculty of Medicine and Health, School of Pharmacy, The University of Sydney, Camperdown, NSW, Australia; Department of Pharmacy, Royal Prince Alfred Hospital, Camperdown, NSW, Australia; St George and Sutherland Clinical School, University of New South Wales, Kogarah, NSW, Australia; School of Clinical Medicine, University of New South Wales, Kensington, NSW, Australia; School of Medicine, University of Notre Dame, Chippendale, NSW, Australia; South Western Sydney Clinical School, Faculty of Medicine, UNSW Sydney, Liverpool, NSW, Australia; Rheumatology Department, Liverpool Hospital, SWSLHD, Liverpool, NSW, Australia; Maridulu Budyari Gumal, Sydney Partnership of Health Education Research and Enterprise (SPHERE), Sydney, NSW, Australia; Faculty of Medicine and Health, School of Pharmacy, The University of Sydney, Camperdown, NSW, Australia; Department of Pharmacy, Prince of Wales Hospital, Randwick, NSW, Australia; Whitlam Orthopaedic Research Centre, Ingham Institute, Liverpool, NSW, Australia; South Western Sydney Clinical School, Faculty of Medicine, UNSW Sydney, Liverpool, NSW, Australia

**Keywords:** Subacute Pain, Total Hip Arthroplasty, Total Knee Arthroplasty, Intervention, Joint Replacement

## Abstract

**Background:**

Total knee arthroplasty (TKA) and total hip arthroplasty (THA) surgeries are among the most common elective procedures. Moderate to severe postoperative pain during the subacute period (defined here as the period from hospital discharge to 3 months postoperatively) is a predictor of persistent pain 12 months postoperatively. This review aimed to examine the available postdischarge pharmacological interventions, including educational and prescribing strategies, and their effect on reducing pain during the subacute period after TKA or THA.

**Methods:**

We searched seven electronic databases from inception to April 22, 2021. Published randomized controlled trials of adults who underwent TKA or THA and received a pharmacological-based intervention commencing within 1 week after hospital discharge and conducted for up to 3 months postoperatively were compared with any treatment. Two reviewers independently extracted data on the primary outcome, pain intensity. This review was registered prospectively on PROSPERO (ID: CRD42021250384).

**Results:**

Four trials involving 660 participants were included. Interventions included changing analgesic prescribing practices upon hospital discharge and education on analgesic use. Providing multimodal non-opioid analgesia in addition to reduced opioid quantity was associated with lower subacute pain (coefficient –0.81; 95% confidence interval –1.33 to –0.29; *P* = 0.003). Education on analgesic use during multidisciplinary home visits was effective for reducing pain intensity during the subacute period (6.25 ± 10.13 vs 35.67 ± 22.05; *P* < 0.001) compared with usual care.

**Conclusions:**

Interventions involving the provision of multimodal non-opioid analgesia and education on analgesic use show positive effects on reducing pain intensity during the subacute period after TKA and THA.

## Background

Total knee arthroplasty (TKA) and total hip arthroplasty (THA) are cornerstone and cost-effective procedures to improve pain, mobility, and quality of life for people with severe knee and hip osteoarthritis, respectively [1, 2]. TKA and THA are among the most common elective surgeries performed worldwide [3–5]. Due to rising rates of obesity and population aging, the burden of osteoarthritis and the subsequent need for these surgeries are projected to increase by 673% for TKA and 174% for THA by 2030 in the United States (US) [6], with similar increases projected elsewhere, such as in Australia [5] and the United Kingdom [4].

Adequate pain management after TKA and THA facilitates faster rehabilitation and reduced postoperative complications, hospital readmission rates, and overall health care costs [7]. A prospective study of 87 patients showed that moderate to severe subacute pain is experienced by more than 40% of patients and is associated with an increased risk of persistent pain 12 months after orthopedic surgery [8]; thus, it would appear that better early management could confer better long-term results. Although there is some literature describing pain management strategies in the immediate postoperative period after arthroplasty [9, 10], the most effective strategies for the management of pain during the subacute period (from discharge to 3 months after surgery) are unclear [8, 11–13]. Uncertainty about the optimal strategy might contribute toward unwarranted practice variation and low-value or harmful care, which can adversely impact patient outcomes.

The most effective pharmacological-based interventions for subacute pain after TKA or THA have not been established, which represents a gap in evidence for providing care in the subacute postoperative period. Therefore, the present systematic review will examine the available evidence for postdischarge pharmacological interventions, including educational and prescribing strategies, and their effect on reducing pain during the subacute period after TKA or THA.

## Methods

This review was performed in adherence to the Preferred Reporting Items for Systematic Reviews and Meta-Analyses (PRISMA) guidelines [14]. This review was prospectively registered on PROSPERO (ID: CRD42021250384).

### Inclusion and Exclusion Criteria

#### Types of Studies

We included original peer-reviewed randomized controlled trials (RCTs) written in English. We excluded all other study types and conference abstracts.

#### Types of Participants

Studies with adult participants (18 years of age or older) who had undergone primary TKA or THA were included. Studies in which analgesics were used exclusively for palliative care, opioid-substitution therapy, or cancer-related pain were excluded, as they were outside the scope of this review.

#### Types of Interventions

The effectiveness of exercise-based rehabilitation interventions on pain outcomes after TKA or THA has been explored in several systematic reviews. No physiotherapy-based exercise intervention has been found to be clinically superior to another for pain outcomes in the subacute phase after surgery [15–17]. Thus, we targeted studies focusing on pharmacological-based interventions commencing within 1 week after hospital discharge that aimed to reduce index joint pain and were conducted for up to 3 months after TKA or THA. Pharmacological interventions may have involved any intervention to optimize pharmacological therapy and included educational and prescribing strategies relating to medication use. The comparator group(s) included any strategy, including medication, exercise programs, biopsychosocial, alternative medicine (e.g., acupuncture), interventional procedures, and/or usual care.

#### Types of Outcome Measures

We extracted relevant measures before and after the intervention, up to 3 months postoperatively. The primary or secondary outcome of the study must have included index joint pain up to 3 months postoperatively.

The primary outcome of this review was index joint pain intensity up to 3 months after TKA or THA.

Secondary outcomes included postoperative overall body pain, analgesic use (including opioid use in morphine milligram equivalents [MMEs]), incidence of adverse events, physical function, length of hospital stay, hospital readmission rate, psychological functioning, disease-specific function or quality of life, and overall quality of life collected up to 12 months after surgery. We also collected information on how studies defined the subacute period after surgery.

These criteria are shown in the Population, Intervention, Comparison, Outcome (PICO) format in Supplementary Data Table S1.

### Search Strategy

We conducted a systematic search in seven electronic databases: Medline (1960–present), Scopus (1960–present), Embase (1969–present), Cochrane Central Register of Controlled Trials (1995–present), International Pharmaceutical Abstracts (1970–present), PsycINFO (1963–present), and the Cumulative Index of Nursing and Allied Health Literature (CINAHL, 1937-present). The search was conducted from database inception to April 22, 2021.

The search terms applied to all electronic databases were developed with an academic librarian and integrated three key filters: postoperative pain management; hip or knee replacement surgery or arthroplasty; and RCTs. The same key terms were applied across all databases with appropriate syntax and subject headings. The full search strategy is available in Supplementary Data Table S2.

References of relevant articles were screened to identify additional studies not captured by the search strategy. Where required, we contacted the authors of potentially eligible articles to obtain additional data relevant to this review and not present in the published articles. The gray literature was also searched via Proquest Dissertations and Theses.

### Data Extraction and Analysis

#### Selection of Studies

After the removal of duplicates, two authors (SL and FG) independently filtered articles by title and abstract for potentially eligible studies. Full-text articles were then assessed independently by the same two authors to confirm eligibility. Any discrepancies were determined by consensus with other team members (JN, JP, and AEP).

#### Data Extraction and Management

Two authors (SL and FG) independently extracted data using a standard data extraction form (Supplementary Data Table S3) that included details of participants, study design, intervention method and duration, and treatment outcomes. Discrepancies were discussed with other team members (JN, JP, and AEP) as required.

#### Assessment of Risk of Bias in the Included Studies

Quality assessment of all included studies was conducted. Four authors (SL, JN, AEP, and JP) were involved in this process and used the Revised Cochrane Risk of Bias Assessment Tool for Randomized trials (RoB 2) [18]. Five domains (random sequence generation, allocation concealment, blinding, incomplete outcome data, and selective reporting) were used to categorize RCTs as possessing low, high, or some concern of risk of bias.

#### Data Synthesis

Heterogeneity among studies was assessed by comparing study design, intervention approach, and outcomes. Because of heterogeneity in interventions and outcomes among studies, a meta-analysis could not be performed. Thus, a narrative synthesis of available studies according to intervention type and our primary and secondary outcomes was conducted.

#### Quality of the Evidence for the Primary Outcome

We planned to conduct a Grading of Recommendations, Assessment, Development, and Evaluations (GRADE) analysis to assess the certainty of the evidence for the primary outcome using guidelines outlined in the *Cochrane Handbook for Systematic Reviews of Interventions* [19].

## Results

**Figure 1.**
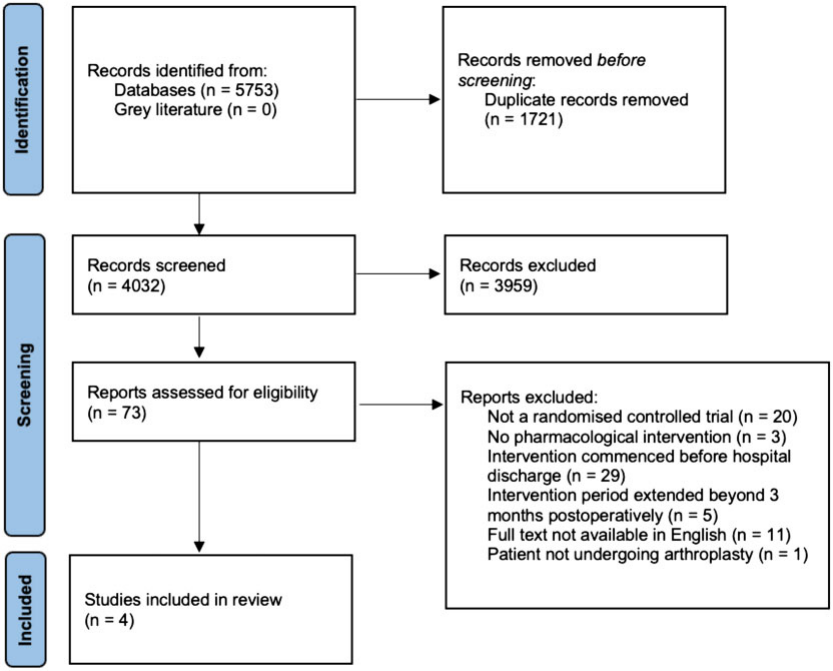
Study inclusion and exclusion criteria flow diagram.

The search strategy generated a total of 5,753 articles, of which 73 full-text articles were assessed for eligibility. Refinement by the inclusion and exclusion criteria resulted in four studies [19–22] being eligible for inclusion (Figure 1).

### Study Characteristics

One study was a three-arm, parallel-group, cluster RCT [19], and the remaining three studies were two-arm RCTs [20–22]. In all studies, the intervention commenced upon hospital discharge, and participants were followed up for 1–3 months. Two studies included patients undergoing TKA only [21, 22], one study included patients undergoing THA only [19], and one study included patients undergoing both THA and TKA [20].

### Risk of Bias in Included Studies

**Figure 2.**
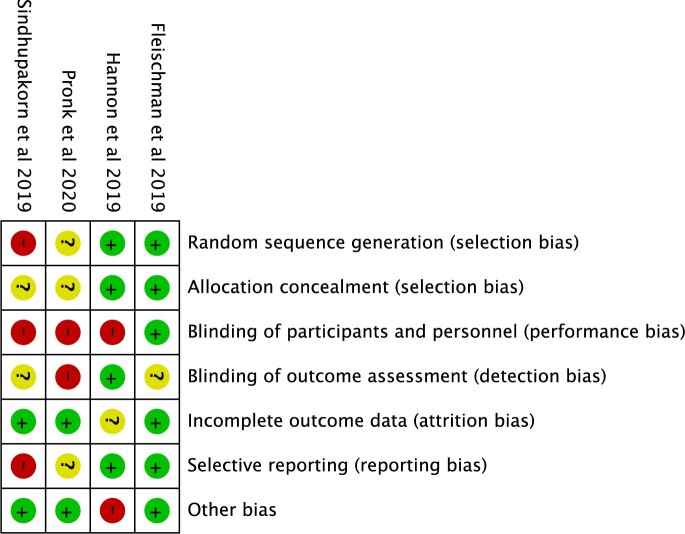
Risk-of-bias summary: Authors’ judgments about each risk-of-bias item for the included studies. Risk of Bias: + = Low; − = High; ? = Unclear.

Two of the four included studies [19, 20] used appropriate methods for random sequence generation and allocation concealment (Figure 2). Blinding of participants and personnel was not achieved in three studies [20–22] because of the nature of the interventions. One study [20] blinded outcome assessors to treatment allocation, two studies [19, 22] did not provide sufficient information, and one study [21] did not blind outcome assessors. Some concerns for attrition bias due to incomplete outcome data secondary to loss to participant follow-up existed in one study [20]. Two studies [19, 20] minimized selective outcome reporting by prospectively registering study protocols in a trial registry. Other risk of bias due to deviation from the intended intervention existed in one study [20]. The full risk-of-bias assessments are available in Supplementary Data Table S4.

### Quality of the Evidence for the Primary Outcome

Because of significant variation in the interventions used and outcome measures between studies, a GRADE analysis was not undertaken.

### Types of Interventions

Studies used interventions that could be classified into two categories: 1) prescribing interventions to change analgesic prescribing practices upon hospital discharge (n = 2) [19, 20], and 2) patient education on analgesic use during the subacute period (n = 2) [21, 22]. Of the educational interventions, one study provided patient-specific advice through a mobile phone application [21]. Another study provided patient education during multidisciplinary patient home visits [22] (Table 1).

**Table 1.** Summary of included RCTs reporting pharmacological interventions to reduce subacute pain after TKA or THA

Author, Year, Country, Funding	Study Size (n; Intervention, Control), Study Design	Study Population, Intervention Commencement and Duration, Follow-Up Duration	Intervention Group(s)	Comparator Group(s)	Outcomes
Fleischman et al., 2019, USA [19], Nil declared sources of funding.	235; Group A = 77 Group B = 79 Group C = 79, Prospective, three-arm, parallel-group, cluster-randomized trial	Adults undergoing primary unilateral THA, Intervention commenced upon hospital discharge; Intervention duration 4 weeks, Follow-up at 1 month and 3 months	Group A: multimodal with minimal opioid supply: Multimodal regimen involving paracetamol, gabapentin, and meloxicam Two-day opioid supply involving 10 tablets each of oxycodone and tramadol Patient education to use non-opioid analgesia on fixed schedule and opioids only when required.	Group B: multimodal with traditional opioid supply: Multimodal analgesic regimen Two-week supply of opioids involving 60 tablets each of oxycodone and tramadol Patient education to use non-opioid analgesia on fixed schedule and opioids only when required.	Pain intensity VAS pain scores lower for Group A (coefficient –0.81; 95% CI –1.33 to –0.29; *P* = 0.003) and Group B (coefficient –0.61; 95% CI –1.13 to –0.09; *P* = 0.021) than for Group C during the first 30 days postoperatively. No significant difference in pain scores between Group A and Group B (coefficient –0.20; 95% CI –0.72 to 0.33; *P* = 0.46).
				Group C: traditional opioid supply: Paracetamol 60 tablets each of oxycodone and tramadol Patient education to take all medications when required, starting with paracetamol for mild pain and opioid medications for moderate or severe pain.	Analgesic use Daily MME lower for Group A (coefficient –0.77; 95% CI –1.06 to –0.47; *P* < 0.001) and Group B (coefficient –0.30; 95% CI –0.60 to –0.01; *P* = 0.04) than for Group C during the 30 days postoperatively. Daily MME lower for Group A than for Group B (coefficient –0.46; 95% CI –0.76 to –0.17; *P* = 0.002) during the 30 days after hospital discharge. Mean total MME use was 44.8 mg for Group A, 79.9 mg for Group B, and 109.8 mg for Group C during the 30 days postoperatively. Mean time to opioid discontinuation shorter in Group A (1.14 weeks; *P* < 0.001) and Group B (1.39 weeks; *P* = 0.001) than in Group C (2.57 weeks).
					Adverse events Mean composite ORSDS score over 4 weeks for Group A was significantly lower than that for Group C (*P* = 0.005). No difference in ORSDS score between Groups B and C (*P* = 0.13).
					Physical function HOOS function did not differ between groups at 90 days postoperatively (*P* = 0.86).
					Hospital readmissions ED visits occurred two times in Group A (2.6%), three times in Group B (3.8%), and five times in Group C (6.3%, *P* = 0.50). Hospital readmissions occurred three times in Group A (3.9%) and four times in Group C (5.1%).
Hannon et al., 2018, USA [20], Several of the authors disclosed associations with an entity in the biomedical field (may include payment) that could be perceived to have potential conflict of interest with this work.	304; I = 161 C = 143, Prospective, single-center, single-blinded, RCT	Adults undergoing unilateral primary THA or TKA, Intervention commenced upon hospital discharge; Intervention duration single time point at discharge. Follow-up at 30 days, 6 weeks, and 90 days.	Patients received 30 tablets of oxycodone 5 mg upon hospital discharge.	Patients received 90 tablets of oxycodone 5 mg upon hospital discharge.	Pain intensity Median Defense and Veterans Pain Rating Scale score did not differ between oxycodone 30-tablet group (median 2.2 [range 0–6.3]) and oxycodone 90-tablet group (median 2.2, range 0–8.4; *P* = 0.811).
					Analgesic use Mean total MME consumption did not differ between patients given 30 or 90 oxycodone 5-mg tablets upon hospital discharge at 30 days after discharge (455.8 ± 320.9 vs 461.9 ± 387.3; *P* = 0.881). Unused oxycodone tablets were lower among patients given 30 oxycodone tablets upon hospital discharge than among patients given 90 tablets upon discharge (median 15 [range 0–30] vs 73 [0–90]; *P* < 0.001) at 30 days after discharge. Oxycodone 5 mg prescription refills were significantly higher among patients given 30 oxycodone 5-mg tablets than among patients given 90 oxycodone 5-mg tablets (26.7% vs 10.5%; *P* < 0.001) within 90 days of discharge. Tramadol prescription refills did not differ significantly between groups (48.4% in the 30 OxyIR group vs 38.8% in the 90 OxyIR group; *P* = 0.13).
Pronk et al., 2020, The Netherlands [21], Nil declared sources of funding.	71; I = 38 C = 33, Single-center, unblinded RCT	Adults undergoing primary TKA, Intervention commenced upon hospital discharge; Intervention duration 2 weeks, Follow-up at 1 month.	Usual care plus PainCoach mobile phone app involving pain level patient input, personalized advice on pain medication use, physiotherapy exercises including videos, use of ice or heat packs, rest, immobilization of leg, and when to call the clinic.	Usual care involving postoperative medication, group information meetings, information booklet, and 24/7 clinic to answer questions.	Pain intensity VAS pain scores did not differ significantly between the intervention and control groups at rest (median 11.5 [IQR 5.0–20.8] vs 10.0 [5.0–25.0]; *P* = 0.77), during activity (14.0 [7.0–28.8] vs 15.0 [8.0–35.0]; *P* = 0.49) or at night (15.0 [7.0–33.0] vs 15.0 [7.0–27.8]; *P* = 0.79) at 1 month after surgery. VAS pain score reduction among active PainCoach use subgroup (total app use at least 12 times; n = 12) was 4.1 times faster during activity compared with control (95% CI –7.5 to –0.8; *P* = 0.02).
					Analgesic use Opioid use was 23.2% lower in PainCoach group (95% CI –38.3 to –4.44; *P* = 0.02) than in controls. Paracetamol use was 14% higher in PainCoach group (95% CI 8.2 to 21.3; *P* < 0.01) than in controls. Analgesic use among active PainCoach app use subgroup involved 44.3% less opioid use (95% CI –59.4 to –23.5; *P* < 0.01), 76.3% less gabapentin use (95% CI –86.0 to –59.8; *P* < 0.01), and 21% more paracetamol use (95% CI 12.6 to 30.0; *P* < 0.01) compared with controls.
					Physical function KOOS-PS was significantly lower in the active PainCoach subgroup than in controls at 1 month postoperatively (mean 33.5 [SD 8.4] vs 39.6 [SD 9.8]; *P* = 0.048). OKS did not vary between intervention and control groups (mean 28.4 [8.4] vs 26.8 [6.2]; *P* = 0.42).
					Quality of life EQ-5D-3L scores did not vary between intervention and control groups (median 80.0 [IQR 70.0–90.0] vs 80.0 [65.5–89.5]; *P* = 0.56).
Sindhupakorn et al., 2019, Thailand [22], Nil declared sources of funding.	50; I = 25 C = 25, RCT	Adults undergoing TKA, Intervention commenced upon hospital discharge; Intervention duration 6 weeks, Follow-up at 2 weeks and 6 weeks	Two home visits within 6 weeks of hospital discharge. Home visits involvement assessment of patient and family aspects using acronym INHOMESSS; I= immobility, N= nutrition of patient, H= home environment, O= other people, M= medications, E= examination, S= spiritual, S= service, S= safety. Each factor was assessed during home visit by a surgeon, nurses, physiotherapists, and a nutritionist, and corrective recommendations were made where required.	Usual care	Analgesic use VAS pain scores were lower among intervention group than among controls at 6 weeks after surgery (6.25 ± 10.13 vs 35.67 ± 22.05; *P* < 0.001).
					Physical function WOMAC scores were higher among intervention group than among controls (88.29 ± 10.66 vs 68.00 ± 12.47; *P* < 0.001). Knee Society Scores were higher among intervention group than among controls (81.67 ± 10.08 vs 68.38 ± 6.45; *P* < 0.001). Knee Society Function Scores were higher among intervention group than among controls (77.83 ± 4.22 vs 73.70 ± 7.48; *P* = 0.037). Knee joint range of motion was greater among intervention group than among controls (107.71 ± 8.47 degrees during extension-flexion vs 98.17 ± 9.57 degrees during extension-flexion; *P* = 0.001). Time until independent patient mobilization was reduced among intervention group compared with control (2.75 ± 0.99 weeks vs 3.71 ± 1.23 weeks; *P* = 0.005).

I= intervention; C= control; USA= United States of America; RR= risk reduction; IQR= interquartile range; SD= standard deviation ; ORSDS= Opioid-Related Symptom Distress Scale; HOOS= Hip Disability and Osteoarthritis Outcome Score; ED= emergency department; KOOS-PS= Knee Injury and Osteoarthritis Outcome Score—Physical Function Short-Form; OKS= Oxford Knee Score; OxyIR= Oxycodone Immediate Release; EQ-5D-3L= EuroQol-5 Dimensions 3-level version; WOMAC= Western Ontario and McMaster Universities Arthritis Index.

#### Prescribing Interventions

Two studies examined the effect of analgesic prescribing interventions at hospital discharge on subacute pain levels after TKA or THA [19, 20]. A three-arm cluster-RCT of 235 patients conducted by Fleischman et al. [19] randomized primary THA patients to receive one of three analgesia plans upon hospital discharge. Patients randomized to Group A received multimodal non-opioid analgesia (paracetamol 1,000 mg every 8 hours, gabapentin 200 mg every 12 hours, meloxicam 15 mg daily) and 10 tablets each of oxycodone and tramadol. Group B patients received the same multimodal non-opioid analgesics and 60 tablets each of oxycodone and tramadol. Patients in Groups A and B were instructed to take non-opioid analgesics on a regular basis and opioids only when required. Finally, Group C patients received only paracetamol and 60 tablets each of oxycodone and tramadol and were instructed to take all medications when required, starting with paracetamol for mild pain and opioids for moderate to severe pain. Lower visual analog scale (VAS) pain scores during the first 30 days after surgery were reported among Group A patients (coefficient –0.81; 95% confidence interval [CI] –1.33 to –0.29; *P* = 0.003) and Group B patients (coefficient –0.61; 95% CI –1.13 to –0.09; *P* = 0.021) than in the Group C cohort. No significant difference in VAS pain scores was identified between Groups A and B (coefficient –0.20; 95% CI –0.72 to 0.33; *P* = 0.46). Significantly lower daily MME opioid use was reported in Group A patients (coefficient –0.77; 95% CI –1.06 to –0.47; *P* < 0.001) and Group B patients (coefficient –0.30; 95% CI –0.60 to –0.01; *P* = 0.04) than in Group C patients during the 30-day postoperative period. During this period, patients allocated to Group A also reported lower daily MME opioid use than did the Group B cohort (coefficient –0.46; 95% CI –0.76 to –0.17; *P* = 0.002). The average MME opioid use in total was 44.8 mg among Group A patients, 79.9 mg among Group B patients, and 109.8 mg among Group C patients at 30 days postoperatively. The time to opioid discontinuation was also shorter in Group A (1.14 weeks; *P* < 0.001) and Group B (1.39 weeks; *P* = 0.001) than in Group C (2.57 weeks). Adverse events were assessed with the Opioid-Related Symptom Distress Scale. Patients in Group A reported significantly lower mean Opioid-Related Symptom Distress Scale scores at 4 weeks postoperatively than did Group C patients (*P* = 0.005). Physical function was assessed with the Hip Disability and Osteoarthritis Outcome Score. No significant between-group differences were reported at 90 days postoperatively (*P* = 0.86) [19].

An RCT conducted by Hannon et al. [20] randomized 304 patients to receive either 30 or 90 oxycodone 5-mg tablets upon hospital discharge after primary THA or TKA. Pain intensity was measured with the Defense and Veterans Pain Rating Scale. The study reported no significant difference in pain intensity at 30 days postoperatively. No significant difference in mean total MME consumption between patients given 30 or 90 oxycodone 5-mg tablets upon hospital discharge was reported at 30 days after discharge (455.8 ± 320.9 mg vs 461.9 ± 387.3 mg; *P* = 0.881). However, the authors reported significantly fewer unused opioid tablets at 30 days after hospital discharge among patients given 30 oxycodone 5-mg tablets upon discharge than among those given 90 oxycodone 5-mg tablets upon discharge (median 15 [range 0–30] vs 73 [range 0–90] tablets; *P* < 0.001). Although more patients given 30 oxycodone 5-mg tablets on discharge requested an oxycodone refill prescription within 90 days of discharge than did those given 90 tablets of oxycodone 5 mg on discharge (26.7% vs 10.5%; *P* < 0.001), no significant between-group difference in the proportion of patients receiving a tramadol refill within 90 days of discharge was reported (*P* = 0.13) [20].

#### Patient Education Interventions

Two studies evaluated the effect of providing tailored patient education after hospital discharge [21, 22]. An RCT conducted by Pronk et al. [21] randomized 71 patients to receive either 1) a personalized pain management smartphone application involving patient pain score input, personalized advice on pain medication use, physiotherapy exercise, and nonpharmacological pain management (n = 38) or 2) usual care (n = 33) for 14 days after hospital discharge after primary TKA. No significant difference in VAS pain scores was reported between the intervention and control groups. However, the authors noted that patients who used the application at least 12 times in total over the 14-day intervention period (n = 19) reported a 4.1 times faster reduction in VAS pain scores during activity compared with controls (95% CI –7.5 to –0.8; *P* = 0.02). Patients allocated to receive the personalized pain management smartphone application used 23.2% less opioids (95% CI –38.3 to –4.44; *P* = 0.02) and 14% more paracetamol (95% CI 8.2 to 21.3; *P* < 0.01) than did control subjects at 14 days after hospital discharge. Physical function was assessed with the Knee Injury and Osteoarthritis Outcome Score—Physical Function Short-Form (KOOS-PS) and Oxford Knee Score. No significant differences in KOOS-PS or Oxford Knee Scores between the total pain management mobile phone application group and controls were reported. However, active use of the pain management mobile application among patients in the intervention group (total application use at least 12 times; n = 19) was associated with significantly lower KOOS-PS scores than those of controls at 1 month postoperatively (33.5 [standard deviation 8.4] vs 39.6 [standard deviation 9.8]; *P* = 0.048). Finally, no significant between-group differences in quality of life measured by the EuroQol-5 Dimensions 3-level version questionnaire were reported at 14 days after hospital discharge [21].

An RCT conducted by Sindhupakorn et al. [22] compared an intervention involving patient home visits by a multidisciplinary team (including a surgeon, nurses, physiotherapists, and a nutritionist) to optimize the home environment and pain medication use by the patient and their family over two home visits during a 6-week period after hospital discharge after TKA (n = 25) with usual care (n = 25). Patients who received home visits reported significantly reduced VAS pain scores compared with the control group (6.25 ± 10.13 vs 35.67 ± 22.05; *P* < 0.001). This study also assessed physical function with the Knee Society Score, knee joint range of motion, and time until the patient could move independently. Patients who received home visits from a multidisciplinary team after TKA reported significantly higher Knee Society Scores (81.67 ± 10.08 vs 68.38 ± 6.45; *P* < 0.001), higher Knee Society Functional Scores (77.83 ± 4.22 vs 73.70 ± 7.48; *P* = 0.037), greater knee joint range of motion (107.71 ± 8.47 degrees during extension-flexion vs 98.17 ± 9.57 degrees during extension-flexion; *P* = 0.001), and reduced time until the patient could move independently (2.75 ± 0.99 weeks vs 3.71 ± 1.23 weeks; *P* = 0.005) at 6 weeks postoperatively compared with those allocated to the control group [22].

## Discussion

### Summary of Main Findings

This systematic review identified four trials involving 660 randomized participants in which a pharmacological-based intervention, including educational or prescribing strategies, was conducted during the postdischarge (subacute) period and was tested against usual care for the reduction of subacute pain after TKA or THA. Interventions included changing analgesic prescribing practices upon hospital discharge and providing education on analgesic use during the subacute period through the use of mobile phone applications or during multidisciplinary home visits. Reducing the quantity of opioid analgesics supplied on hospital discharge did not lead to worse subacute pain levels [20]. Furthermore, providing additional multimodal non-opioid analgesia (paracetamol, gabapentin, meloxicam) was associated with reduced subacute pain [19]. Education on medication use provided through a personalized mobile application did not significantly impact subacute pain intensity [21]. However, patients receiving education on medication use during multidisciplinary home visits reported reduced pain during the subacute period [22]. The overall quality of the evidence was low, with one trial [19] showing some risk of bias and three trials showing a high risk of bias [20–22]. This was largely due to the inability to blind patients to their treatment allocation or blind outcome assessors, given the nature of the interventions. Because of significant heterogeneity in trial designs, interventions used, and outcome variables across studies, no meta-analysis was performed.

### Comparison with Other Reviews

Previous systematic reviews of interventions to reduce pain after TKA or THA have focused largely on acute or chronic pain, with limited research targeting the subacute period. A systematic review by Fischer et al. conducted in 2005 reported that effective interventions for the reduction of immediate postsurgical pain after THA included peripheral nerve block, intrathecal analgesia, and multimodal non-opioid analgesia [10]. A systematic review on acute pain management after TKA reported similar recommendations [23]. After hospital discharge, however, parenteral analgesic routes might have a limited role in pain management after TKA or THA. A systematic review of RCTs conducted by Wylde and colleagues in 2018 identified that interventions commenced within 3 months postoperatively to reduce chronic pain at 12 months or longer after TKA included physiotherapy, nurse-led, neuromuscular electrical stimulation, and multidisciplinary interventions. Existing literature on interventions conducted during the subacute period predominantly focuses on rehabilitation or exercise-based strategies to reduce pain and/or improve physical function after THA or TKA [15–17, 24]. There remains a paucity of literature summarizing the available pharmacological interventions to reduce subacute pain after THA or TKA. Our systematic review adds to the limited literature by providing evidence that subacute pain can be reduced by improving the judicious use of analgesics upon hospital discharge and providing medication-related education after discharge. Our review indicates that interventions used for managing acute and chronic pain, such as the provision of multimodal analgesia and multidisciplinary care, also appear to be relevant for the subacute period [25]. In particular, our findings reinforce existing literature on the use of nonsteroidal anti-inflammatory drugs for their opioid-sparing effect [26] and improved pain relief upon rest and movement after orthopedic surgery [27, 28], provided clinical precautions are addressed. However, although low-dose gabapentin was used in the multimodal protocol by Fleischman et al. [19], the literature does not support the use of gabapentinoids for postoperative pain because of the lack of a clinically significant difference in pain and an association with a higher incidence of adverse events [29]. This represents an opportunity for practice improvement to ensure the judicious use of multimodal analgesia during the subacute postoperative period.

Educational interventions are often used for the management of musculoskeletal pain and thus also appear to be relevant during the subacute phase. The studies exploring educational interventions by Pronk et al. [21] and Sindhupakorn et al. [22] emphasize the importance of patient and clinician education alongside the provision of analgesia to facilitate the safe and appropriate use of medications given upon the transition of care during hospital discharge. Consistency in the management of pain across the acute, subacute, and chronic periods facilitates enhanced continuity of care and, in turn, is known to lead to improved health outcomes, higher patient satisfaction, and more cost-effective care [30].

### Future Considerations

Definitions of the subacute period vary widely in the literature, ranging from 30 days [12] to 90 days after hospital discharge [8, 11]. This variation presents a challenge in the generalizability of different interventions conducted in the subacute period reported across the literature. Furthermore, the lack of a clear and consistent definition of the subacute period might contribute toward ambiguity about the appropriate management of pain during this period. Given that subacute pain is experienced by more than 40% of patients after orthopedic surgery and is associated with an increased risk of persistent pain at 12 months postoperatively [8], adequate management of subacute pain could confer improved quality of life and long-term improvements in pain outcomes. Thus, future studies should ensure explicit reporting of the time period respective to surgery in which interventions were conducted to allow accurate conclusions on appropriate subacute pain management to be reached.

### Strengths and Limitations

To our knowledge, this is the first review to examine the effect of pharmacological-based interventions on reducing pain during the subacute period after TKA or THA. We prospectively registered and adhered to the protocol of this systematic review. Only RCTs were included to improve the reliability of our findings.

However, there are several limitations to this study. Although we conducted a rigorous search across seven electronic databases, reference lists of included studies, and the gray literature, the search strategy was limited to articles written in the English language. Thus, relevant articles in other languages may not have been identified. We were unable to conduct a meta-analysis because of the heterogeneous nature of the included studies. As the number of included trials was relatively small, we were also unable to assess for publication bias with funnel plots [31]. Finally, most included studies showed a high risk of bias. This reduces the confidence that the review findings reflect the true treatment effect of each intervention.

## Conclusion

Interventions involving the provision of multimodal non-opioid analgesia, a lower quantity of opioid analgesics, and patient education on analgesic use appear to be effective strategies to reduce pain intensity during the subacute period after TKA and THA. Further high-quality randomized controlled studies with rigorous and comparable study designs are needed to expand on, and quantitatively synthesize, the existing and any newly emerging data.

## Supplementary Material

pnac052_Supplementary_Data

## Notes

Funding sources: This study was partly funded by the Musculoskeletal Clinical Academic Group (MSK CAG) of the Sydney Partnership for Health, Education, Research and Enterprise (SPHERE). Shania Liu is supported by a National Health and Medical Research Council (NHMRC) Postgraduate Scholarship. These funding sources had no role in the design of the study, data collection and analysis, or preparation of the manuscript.

Authors Jonathan Penm and Justine Naylor contributed equally to this work as co–senior authors.

Study registration: PROSPERO ID: CRD42021250384.
