# Supplementary material for: Effectiveness of Pharmacological-Based Interventions, Including Education and Prescribing Strategies, to Reduce Subacute Pain After Total Hip or Knee Arthroplasty: A Systematic Review of Randomized Controlled Trials
Source: Pain Med. 2022 Mar 24;23(9):1476–88. doi: 10.1093/pm/pnac052 (PMC9434276; doi:10.1093/pm/pnac052)
Supplement: pnac052_Supplementary_Data [file pnac052_supplementary_data.zip › pnac052_Supplementary_Data/Subacute Pain SR Supplementary Material 18.03.22_Clean.docx]

**Supplementary Material**

**Effectiveness of pharmacological interventions to reduce subacute pain following total hip or knee arthroplasty: a systematic review of randomised controlled trials**

Shania Liu, Furkan Genel, Jonathan Penm, Ian Harris, Asad E Patanwala, Sam Adie, Jennifer Stevens, Geraldine Hassett, Kate Luckie, Justine Naylor

**Supplementary Table S1:** Population, Intervention, Comparison, Outcome (PICO) Box

| **Population** | Adults (aged 18 years or older) who had undergone primary total hip or knee arthroplasty |
| --- | --- |
| **Intervention** | At least one pharmacological-based intervention commencing within one week after hospital discharge that aimed to reduce index joint pain. Pharmacological interventions may involve any change or difference in pharmacological therapy and included educational or prescribing strategies relating to medication use. |
| **Comparison** | Any strategy, including medication, exercise programs, biopsychosocial, alternative medicine (e.g. acupuncture), interventional procedures and/or usual care. |
| **Outcome** | The primary outcome was index joint pain intensity up to three months after total hip or knee arthroplasty.  Secondary outcomes included postoperative overall body pain, physical function, analgesic use (including opioid use in morphine milligram equivalents), incidence of adverse events, length of hospital stay, hospital readmission rate, psychological functioning, disease-specific function or quality of life and overall quality of life collected up to 12 months after surgery. We also collected how studies defined the subacute period after surgery. |

**Supplementary Table S2:** Search terms

| MEDLINE (1946 to Present) (OvidSP) |
| --- |
| 1. Total hip replacement.mp. or Arthroplasty, Replacement, Hip/ 2. Total knee replacement.mp. or Arthroplasty, Replacement, Knee/ 3. (Pain adj2 reduc*).mp. 4. (Pain adj2 control*).mp. 5. Pain Management/ 6. (Pain adj2 manag*).mp. 7. Postoperative pain.mp. or Pain, Postoperative/ 8. 1 or 2 9. 3 or 4 or 5 or 6 or 7 10. 8 and 9 11. Randomized Controlled Trials as Topic/ or control* trial*.mp. or Clinical Trials as Topic/ 12. 10 and 11 |

**Supplementary Table S3:** Data extraction form

| **Author, Year, Country,**  **Funding** | **Study size (n; Intervention, Control)** | **Study design** | **Study population,**  **Follow-up duration** | **Intervention group** | **Comparator group(s)** | **Outcomes** |
| --- | --- | --- | --- | --- | --- | --- |
|  |  |  |  |  |  |  |
|  |  |  |  |  |  |  |

**Supplementary Table S4**: Risk of bias comments and judgements

| **Fleischman et al. 2019 [20]** | | | |
| --- | --- | --- | --- |
| **Domain 1a: Risk of bias arising from the randomization process** | **Signalling questions** | **Comments** | **Response options** |
|  | **1a.1 Was the allocation sequence random?** | Yes (used a random number generator at study commencement.)  Allocation sequence was concealed as a random number generator was used. | Y |
|  | **1a.2 Was the allocation sequence concealed until clusters were enrolled and assigned to interventions?** |  | Y |
|  | **1a.3 Did baseline differences between intervention groups suggest a problem with the randomization process?** | No | N |
|  | **Risk-of-bias judgement** | Per algorithm | Low risk |
| **Domain 1b: Risk of bias arising from the timing of identification or recruitment of participants in a cluster-randomized trial** | **1b.1 Were all the individual participants identified and recruited (if appropriate) before randomization of clusters?** | Yes | Y |
|  | **1b.2 If N/PN/NI to 1b.1: Is it likely that selection of individual participants was affected by knowledge of the intervention assigned to the cluster?** |  | NA |
|  | **1b.3 Were there baseline imbalances that suggest differential identification or recruitment of individual participants between intervention groups?** |  | N |
|  | **Risk-of-bias judgement** | Per algorithm | Low risk |
| **Domain 2: Risk of bias due to deviations from the intended interventions (effect of assignment to intervention)** | **2.1a Were participants aware that they were in a trial?** | This study involved allocating surgeons to three analgesic supply schedules. It was deemed not feasible and unethical to achieve blinding. The authors describe authorised deception where each surgeon acted as a ‘guardian’ and allowed each of the interventions to be implemented as standard of care for the allotted time period. Thus the surgeons were aware of treatment allocation but patients may not be aware of their allocation to intervention/comparator groups. | PY |
|  | **2.1b. If Y/PY/NI to 2.1a: Were participants aware of their assigned intervention during the trial?** | Partial yes because the surgeons were aware of their assigned intervention during the trial but patients were not aware of their assignment due to authorised deception. | PY |
|  | **2.2. Were carers and people delivering the interventions aware of participants' assigned intervention during the trial?** |  | PY |
|  | **2.3. If Y/PY/NI to 2.1 or 2.2: Were there deviations from the intended intervention that arose because of the trial context?** |  | N |
|  | **2.4 If Y/PY to 2.3: Were these deviations likely to have affected the outcome?** |  | NA |
|  |  |  |  |
|  | **2.5. If Y/PY/NI to 2.4: Were these deviations from intended intervention balanced between groups?** |  | NA |
|  | **2.6 Was an appropriate analysis used to estimate the effect of assignment to intervention?** |  | Y |
|  | **2.7 If N/PN/NI to 2.6: Was there potential for a substantial impact (on the result) of the failure to analyse participants in the group to which they were randomized?** |  | NA |
|  | **Risk-of-bias judgement** | Per algorithm | Some concerns |
| **Domain 2: Risk of bias due to deviations from the intended interventions (effect of adhering to intervention)** | **2.1. Were participants aware of their assigned intervention during the trial?** | As the patients were the assessors of pain and they were blinded via authorised deception (they believed it was usual care), it is unlikely their assessment was influenced by knowledge of the intervention | N |
|  | **2.2. Were carers and people delivering the interventions aware of participants' assigned intervention during the trial?** |  | N |
|  | **2.3. [If applicable:] If Y/PY/NI to 2.1 or 2.2: Were important non-protocol interventions balanced across intervention groups?** |  | NA |
|  | **2.4. [If applicable:] Were there failures in implementing the intervention that could have affected the outcome?** |  | NA |
|  | **2.5. [If applicable:] Was there non-adherence to the assigned intervention regimen that could have affected participants’ outcomes?** |  | NA |
|  | **2.6. If N/PN/NI to 2.3, or Y/PY/NI to 2.4 or 2.5: Was an appropriate analysis used to estimate the effect of adhering to the intervention?** |  | NA |
|  | **Risk-of-bias judgement** | Per algorithm | Low risk |
| **Domain 3: Risk of bias due to missing outcome data** | **3.1a Were data for this outcome available for all clusters that recruited participants?** | The authors state that linear mixed models and generalised linear mixed models were used to assess patients with missing outcomes data | PY |
|  | **3.1b Were data for this outcome available for all, or nearly all, participants within clusters?** | As above | PY |
|  | **3.2 If N/PN/NI to 3.1a or 3.1b: Is there evidence that the result was not biased by missing data?** |  | NA |
|  | **3.3 If N/PN to 3.2 Could missingness in the outcome depend on its true value?** |  | NA |
|  | **3.4 If Y/PY/NI to 3.3: Is it likely that missingness in the outcome depended on its true value?** |  | NA |
|  | **Risk-of-bias judgement** | Per algorithm | Low risk |
| **Domain 4: Risk of bias in measurement of the outcome** | **4.1 Was the method of measuring the outcome inappropriate?** | The primary outcome was measured using the visual analogue scale and daily IV morphine equivalents which appear appropriate | N |
|  | **4.2 Could measurement or ascertainment of the outcome have differed between intervention groups?** |  | N |
|  | **4.3a If N/PN/NI to 4.1 and 4.2: Were outcome assessors aware that a trial was taking place?** | Not reported but assessors were likely aware that a trial was taking place | NI |
|  | **4.3b If Y/PY/NI to 4.3a: Were outcome assessors aware of the intervention received by study participants?** | Not reported but assessors were likely aware of treatment allocation | NI |
|  | **4.4 If Y/PY/NI to 4.3b: Could assessment of the outcome have been influenced by knowledge of intervention received?** | Not reported, but assessment of the outcome may potentially be influenced by knowledge of the intervention received. Nevertheless, patients were blinded via authorised deception (they believed it was usual care), thus it is unlikely their assessment was influenced by knowledge of the intervention. | PN |
|  | **4.5 If Y/PY/NI to 4.4: Is it likely that assessment of the outcome was influenced by knowledge of intervention received?** |  | PN |
|  | **Risk-of-bias judgement** | Per algorithm | Some concerns |
| **Domain 5: Risk of bias in selection of the reported result** | **5.1 Were the data that produced this result analysed in accordance with a pre-specified analysis plan that was finalized before unblinded outcome data were available for analysis?** | Yes, the trial protocol including a pre-specified analysis plan was published prospectively on ClinicalTrials.gov | Y |
|  | **Is the numerical result being assessed likely to have been selected, on the basis of the results, from...** |  |  |
|  | **5.2. ... multiple eligible outcome measurements (e.g. scales, definitions, time points) within the outcome domain?** | No, one outcome measurement tool was used to assess each outcome | PN |
|  | **5.3 ... multiple eligible analyses of the data?** |  | PN |
|  | **Risk-of-bias judgement** | Per algorithm | Low risk |
| **Overall risk of bias** | **Risk-of-bias judgement** | This study was judged to raise some concerns in at least one domain but did not show high risk of bias for any domain. Thus, per the RoB 2 Guidelines, the overall risk of bias judgement was ‘Some concerns’. | Some concerns |
| **Hannon et al. 2019 [21]** | | | |
| **Domain 1a: Risk of bias arising from the randomization process** | **Signalling questions** | **Comments** | **Response options** |
|  | **1.1 Was the allocation sequence random?** | Yes, the study investigators used blocked randomisation, performed using STATA | Y |
|  | **1.2 Was the allocation sequence concealed until participants were enrolled and assigned to interventions?** |  | Y |
|  | **1.3 Did baseline differences between intervention groups suggest a problem with the randomization process?** | No significant differences between groups in baseline demographics reported, suggesting appropriate randomisation | N |
|  | **Risk-of-bias judgement** |  | Low risk |
| **Domain 2: Risk of bias due to deviations from the intended interventions (effect of assignment to intervention)** | **2.1. Were participants aware of their assigned intervention during the trial?** | Yes, participants were aware of their assigned intervention during the trial (only aware after they were given their discharge oxycodone prescription.)  Clinical staff were blinded to the patients’ treatment group. | PY |
|  | **2.2. Were carers and people delivering the interventions aware of participants' assigned intervention during the trial?** |  | N |
|  | **2.3. If Y/PY/NI to 2.1 or 2.2: Were there deviations from the intended intervention that arose because of the trial context?** | Yes, there were deviations from the intended intervention described in the study. | Y |
|  | **2.4 If Y/PY to 2.3: Were these deviations likely to have affected the outcome?** | Not described | NI |
|  | **2.5. If Y/PY/NI to 2.4: Were these deviations from intended intervention balanced between groups?** | Not described | NI |
|  | **2.6 Was an appropriate analysis used to estimate the effect of assignment to intervention?** | Statistical analysis included t test, rand-sum and Fisher exact tests with alpha = 0.05. | Y |
|  | **2.7 If N/PN/NI to 2.6: Was there potential for a substantial impact (on the result) of the failure to analyse participants in the group to which they were randomized?** |  | NA |
|  | **Risk-of-bias judgement** | Per algorithm | High risk |
| **Domain 2: Risk of bias due to deviations from the intended interventions (effect of adhering to intervention)** | **2.1. Were participants aware of their assigned intervention during the trial?** | Yes, participants were aware of their assigned intervention during the trial (only aware after they were given their discharge oxycodone prescription.)  Clinical staff were blinded to the patients’ treatment group. | Y |
|  | **2.2. Were carers and people delivering the interventions aware of participants' assigned intervention during the trial?** |  | N |
|  | **2.3. [If applicable:] If Y/PY/NI to 2.1 or 2.2: Were important non-protocol interventions balanced across intervention groups?** | Not described | NI |
|  | **2.4. [If applicable:] Were there failures in implementing the intervention that could have affected the outcome?** | Not described | NI |
|  | **2.5. [If applicable:] Was there non-adherence to the assigned intervention regimen that could have affected participants’ outcomes?** | The study investigators report that deviation from the assigned group occurred in 29 instances (90 OxyIR reverted to the default 30 OxyIR due to miscommunication between study staff and prescribers). | PY |
|  | **2.6. If N/PN/NI to 2.3, or Y/PY/NI to 2.4 or 2.5: Was an appropriate analysis used to estimate the effect of adhering to the intervention?** |  | NA |
|  | **Risk-of-bias judgement** | Per algorithm | High risk |
| **Domain 3: Missing outcome data** | **3.1 Were data for this outcome available for all, or nearly all, participants randomized?** | The authors report a total of 114 (27.3%) patients lost to follow up. | PN |
|  | **3.2 If N/PN/NI to 3.1: Is there evidence that the result was not biased by missing outcome data?** |  | NA |
|  | **3.3 If N/PN to 3.2: Could missingness in the outcome depend on its true value?** |  | NA |
|  | **3.4 If Y/PY/NI to 3.3: Is it likely that missingness in the outcome depended on its true value?** |  | NA |
|  | **Risk-of-bias judgement** | Per algorithm | Some concerns |
| **Domain 4: Risk of bias in measurement of the outcome** | **4.1 Was the method of measuring the outcome inappropriate?** | The primary outcome was measured using a 30 day diary, whether physical therapy took place and the validated Defence and Veterans Pain Rating Scale. Thus, these methods were deemed appropriate. | N |
|  | **4.2 Could measurement or ascertainment of the outcome have differed between intervention groups?** | Unlikely | PN |
|  | **4.3 If N/PN/NI to 4.1 and 4.2: Were outcome assessors aware of the intervention received by study participants?** | No, study assessors were blinded to the treatment allocation of participants. | N |
|  | **4.4 If Y/PY/NI to 4.3: Could assessment of the outcome have been influenced by knowledge of intervention received?** |  | NA |
|  | **4.5 If Y/PY/NI to 4.4:** **Is it likely that assessment of the outcome was influenced by knowledge of intervention received?** |  | NA |
|  | **Risk-of-bias judgement** | Per algorithm | Low risk |
| **Domain 5: Risk of bias in selection of the reported result** | **5.1 Were the data that produced this result analysed in accordance with a pre-specified analysis plan that was finalized before unblinded outcome data were available for analysis?** | Yes, the study protocol and pre-specified analysis plan were prospectively published on ClinicalTrials.gov registry. | Y |
|  | **Is the numerical result being assessed likely to have been selected, on the basis of the results, from...** |  |  |
|  | **5.2. ... multiple eligible outcome measurements (e.g. scales, definitions, time points) within the outcome domain?** | One scale per outcome measure was used | N |
|  | **5.3 ... multiple eligible analyses of the data?** |  | N |
|  | **Risk-of-bias judgement** | Per algorithm | Low risk |
| **Overall risk of bias** | **Risk-of-bias judgement** | This study was judged to be at high risk of bias in at least one domain for this result. Thus, per the RoB 2 Guidelines, the overall risk of bias judgement was ‘High risk of bias’ | High risk of bias |
| **Pronk et al. 2020 [22]** | | | |
| **Domain 1a: Risk of bias arising from the randomization process** | **Signalling questions** | **Comments** | **Response options** |
|  | **1.1 Was the allocation sequence random?** | Yes patients were randomly assigned to the PainCoach or control group using sealed opaque envelopes that were only opened upon assignment to intervention.  However, as the lots were 1:1, (one person allocated each group in a 1:1 ratio), the next person’s allocation can be determined and allocation blinding is limited. | Y |
|  | **1.2 Was the allocation sequence concealed until participants were enrolled and assigned to interventions?** |  | PN |
|  | **1.3 Did baseline differences between intervention groups suggest a problem with the randomization process?** | There were no statistically significant differences in baseline demographics between the PainCoach and control groups | N |
|  | **Risk-of-bias judgement** | Per algorithm | Some concerns |
| **Domain 2: Risk of bias due to deviations from the intended interventions (effect of assignment to intervention)** | **2.1. Were participants aware of their assigned intervention during the trial?** | Yes, participants were aware of their allocation during the trial.  The intervention was delivered by an application so this is not applicable. The application, however, was installed on the patient’s phone by an unblinded nurse. | Y |
|  | **2.2. Were carers and people delivering the interventions aware of participants' assigned intervention during the trial?** |  | PY |
|  | **2.3. If Y/PY/NI to 2.1 or 2.2: Were there deviations from the intended intervention that arose because of the trial context?** | None reported | NI |
|  | **2.4 If Y/PY to 2.3: Were these deviations likely to have affected the outcome?** | None reported | NI |
|  | **2.5. If Y/PY/NI to 2.4: Were these deviations from intended intervention balanced between groups?** | None reported | NI |
|  | **2.6 Was an appropriate analysis used to estimate the effect of assignment to intervention?** | Partial – the authors did not specify whether Intention to treat analysis was performed or what was done with missing data. | PN |
|  | **2.7 If N/PN/NI to 2.6: Was there potential for a substantial impact (on the result) of the failure to analyse participants in the group to which they were randomized?** |  | NA |
|  | **Risk-of-bias judgement** | Per algorithm | Some concerns |
| **Domain 2: Risk of bias due to deviations from the intended interventions (effect of adhering to intervention)** | **2.1. Were participants aware of their assigned intervention during the trial?** | Yes, participants were aware of their allocation during the trial.  The intervention was delivered by an application so this is not applicable. The application, however, was installed on the patient’s phone by an unblinded nurse. | Y |
|  | **2.2. Were carers and people delivering the interventions aware of participants' assigned intervention during the trial?** |  | PY |
|  | **2.3. [If applicable:] If Y/PY/NI to 2.1 or 2.2: Were important non-protocol interventions balanced across intervention groups?** | None reported | NI |
|  | **2.4. [If applicable:] Were there failures in implementing the intervention that could have affected the outcome?** | None reported | NI |
|  | **2.5. [If applicable:] Was there non-adherence to the assigned intervention regimen that could have affected participants’ outcomes?** | Only about 50% of the pain coach group participants reported using the application > 12 times in total. | Y |
|  | **2.6. If N/PN/NI to 2.3, or Y/PY/NI to 2.4 or 2.5: Was an appropriate analysis used to estimate the effect of adhering to the intervention?** | Subgroup analysis on the active pain coach application users vs. control was conducted. However, it was unclear whether the defined ‘12’ times of application use was decided a-priori. | Y |
|  | **Risk-of-bias judgement** | Per algorithm | High risk |
| **Domain 3: Missing outcome data** | **3.1 Were data for this outcome available for all, or nearly all, participants randomized?** | Missing outcome data due to loss to follow up was reported in a total of 5 patients and the reasons for loss to follow up were described | Y |
|  | **3.2 If N/PN/NI to 3.1: Is there evidence that the result was not biased by missing outcome data?** |  | NA |
|  | **3.3 If N/PN to 3.2: Could missingness in the outcome depend on its true value?** |  | NA |
|  | **3.4 If Y/PY/NI to 3.3: Is it likely that missingness in the outcome depended on its true value?** |  | NA |
|  | **Risk-of-bias judgement** | Per algorithm | Low risk |
| **Domain 4: Risk of bias in measurement of the outcome** | **4.1 Was the method of measuring the outcome inappropriate?** | The methods of outcome measurement appear appropriate.  Analgesia was patient-reported, which may be subject to under- or over-reporting | PY |
|  | **4.2 Could measurement or ascertainment of the outcome have differed between intervention groups?** | No | N |
|  | **4.3 If N/PN/NI to 4.1 and 4.2: Were outcome assessors aware of the intervention received by study participants?** | The study investigators report that this was an unblinded trial. Outcomes were obtained online. Thus, the investigator conducting statistical analysis may be aware of patient allocation. | PY |
|  | **4.4 If Y/PY/NI to 4.3: Could assessment of the outcome have been influenced by knowledge of intervention received?** | It is likely that the outcome assessors were unblinded and were thus aware of the intervention received which may influence the assessment of outcomes. | PY |
|  | **4.5 If Y/PY/NI to 4.4: Is it likely that assessment of the outcome was influenced by knowledge of intervention received?** |  | PY |
|  | **Risk-of-bias judgement** | Per algorithm | High risk |
| **Domain 5: Risk of bias in selection of the reported result** | **5.1 Were the data that produced this result analysed in accordance with a pre-specified analysis plan that was finalized before unblinded outcome data were available for analysis?** | Potential no as this trial was registered on ClinicalTrials.gov retrospectively. It was unclear whether the active application use group vs. regular application group cut-off of 12 times was defined a-priori.  No primary endpoint was pre-specified. Data appear to be averaged from multiple time points. | N |
|  | **Is the numerical result being assessed likely to have been selected, on the basis of the results, from...** |  |  |
|  | **5.2. ... multiple eligible outcome measurements (e.g. scales, definitions, time points) within the outcome domain?** |  | N |
|  | **5.3 ... multiple eligible analyses of the data?** |  | N |
|  | **Risk-of-bias judgement** | Per algorithm | Some concerns |
| **Overall risk of bias** | **Risk-of-bias judgement** | This study was judged to be at high risk of bias in at least one domain for this result. Thus, per the RoB 2 Guidelines, the overall risk of bias judgement was ‘High risk of bias’ | High risk of bias |
| **Sindhupakorn et al. 2019 [23]** | | | |
| **Domain 1a: Risk of bias arising from the randomization process** | **Signalling questions** | **Comments** | **Response options** |
|  | **1.1 Was the allocation sequence random?** | Simple randomisation was used, but no further details provided.  No information on whether the allocation sequence was concealed until participant enrolment. | NI |
|  | **1.2 Was the allocation sequence concealed until participants were enrolled and assigned to interventions?** |  | NI |
|  | **1.3 Did baseline differences between intervention groups suggest a problem with the randomization process?** | Yes, participants in the TKR-home group had a differences in sex and significantly lower pain score at baseline compared with the TKR group | PY |
|  | **Risk-of-bias judgement** | Per algorithm | High |
| **Domain 2: Risk of bias due to deviations from the intended interventions (effect of assignment to intervention)** | **2.1. Were participants aware of their assigned intervention during the trial?** | Yes the patients and people delivering the intervention would be aware of the allocated assignment as this was an open label study | Y |
|  | **2.2. Were carers and people delivering the interventions aware of participants' assigned intervention during the trial?** |  | Y |
|  | **2.3. If Y/PY/NI to 2.1 or 2.2: Were there deviations from the intended intervention that arose because of the trial context?** | No information regarding adherence of home visits to the guideline INHOMESSS | NI |
|  | **2.4 If Y/PY to 2.3: Were these deviations likely to have affected the outcome?** | NA | NI |
|  | **2.5. If Y/PY/NI to 2.4: Were these deviations from intended intervention balanced between groups?** | NA | NI |
|  | **2.6 Was an appropriate analysis used to estimate the effect of assignment to intervention?** | Follow up missing for 2 patients. Analysis was per protocol rather than ITT | N |
|  | **2.7 If N/PN/NI to 2.6: Was there potential for a substantial impact (on the result) of the failure to analyse participants in the group to which they were randomized?** | Only 2 patients missing. Unlikely to have an impact. | PN |
|  | **Risk-of-bias judgement** | Per algorithm | Some concerns |
| **Domain 2: Risk of bias due to deviations from the intended interventions (effect of adhering to intervention)** | **2.1. Were participants aware of their assigned intervention during the trial?** | Yes the patients and people delivering the intervention would be aware of the allocated assignment as this was an open label study | Y |
|  | **2.2. Were carers and people delivering the interventions aware of participants' assigned intervention during the trial?** |  | Y |
|  | **2.3. [If applicable:] If Y/PY/NI to 2.1 or 2.2: Were important non-protocol interventions balanced across intervention groups?** | No information on this | NI |
|  | **2.4. [If applicable:] Were there failures in implementing the intervention that could have affected the outcome?** | No information on this | NI |
|  | **2.5. [If applicable:] Was there non-adherence to the assigned intervention regimen that could have affected participants’ outcomes?** | No information on this | NI |
|  | **2.6. If N/PN/NI to 2.3, or Y/PY/NI to 2.4 or 2.5: Was an appropriate analysis used to estimate the effect of adhering to the intervention?** | No analysis done | NI |
|  | **Risk-of-bias judgement** | Per algorithm | High risk |
| **Domain 3: Missing outcome data** | **3.1 Were data for this outcome available for all, or nearly all, participants randomized?** | Yes, data on a total of 2 patients lost to follow up were reported and the reasons for loss to follow up were given. | Y |
|  | **3.2 If N/PN/NI to 3.1: Is there evidence that the result was not biased by missing outcome data?** | NA | NA |
|  | **3.3 If N/PN to 3.2: Could missingness in the outcome depend on its true value?** | NA | NA |
|  | **3.4 If Y/PY/NI to 3.3: Is it likely that missingness in the outcome depended on its true value?** |  | NA |
|  | **Risk-of-bias judgement** | Per algorithm | Low risk |
| **Domain 4: Risk of bias in measurement of the outcome** | **4.1 Was the method of measuring the outcome inappropriate?** | Methods for outcome measurement appear appropriate.  Blinding may not be possible based on study design. | PN |
|  | **4.2 Could measurement or ascertainment of the outcome have differed between intervention groups?** | No separation / blinding of allocation to those who performed outcome assessment | PY |
|  | **4.3 If N/PN/NI to 4.1 and 4.2: Were outcome assessors aware of the intervention received by study participants?** | Assessors were not blinded | Y |
|  | **4.4 If Y/PY/NI to 4.3: Could assessment of the outcome have been influenced by knowledge of intervention received?** | It is likely that the outcome assessors were unblinded and were thus aware of the intervention received which may influence the assessment of outcomes (surgeons visited homes) | PY |
|  | **4.5 If Y/PY/NI to 4.4: Is it likely that assessment of the outcome was influenced by knowledge of intervention received?** |  | PY |
|  | **Risk-of-bias judgement** | Per algorithm | High risk |
| **Domain 5: Risk of bias in selection of the reported result** | **5.1 Were the data that produced this result analysed in accordance with a pre-specified analysis plan that was finalized before unblinded outcome data were available for analysis?** | No information about a pre-specified plan | NI |
|  | **Is the numerical result being assessed likely to have been selected, on the basis of the results, from...** |  |  |
|  | **5.2. ... multiple eligible outcome measurements (e.g. scales, definitions, time points) within the outcome domain?** | This are multiple outcomes. No pre-specified primary outcome | PY |
|  | **5.3 ... multiple eligible analyses of the data?** | Measurement occurred at only one time point. No dichotomization of outcome variables. Multiple analysis of same outcome unlikely. | PN |
|  | **Risk-of-bias judgement** | Per algorithm | High risk |
| **Overall risk of bias** | **Risk-of-bias judgement** | This study was judged to be at high risk of bias in at least one domain for this result. Thus, per the RoB 2 Guidelines, the overall risk of bias judgement was ‘High risk of bias’ | High risk of bias |
